# The Efficacy of Triphala in the Management of Minor Aphthous Ulcers: A Case Report

**DOI:** 10.7759/cureus.65404

**Published:** 2024-07-26

**Authors:** Aakanksha V Tiwari, Suwarna Dangore-Khasbage

**Affiliations:** 1 Oral Medicine and Radiology, Sharad Pawar Dental College and Hospital, Datta Meghe Institute of Higher Education and Research, Wardha, IND

**Keywords:** triphala, stress, management, efficacy, aphthous ulcer

## Abstract

Aphthous ulcers, also known as canker sores, are the most frequently encountered lesions in the oral cavity by clinicians and particularly by dentists. It might affect populations of all age groups, although common in the younger age group. Though multifactorial causes are known to be associated with the occurrence of aphthae, the most common etiologies are stress, inadequate sleep, and improper digestion. They can appear on the oral mucosa, palate, gingiva, labial mucosa, and tongue. They can be very uncomfortable during mastication, speech, and deglutition. Generally, the management relies on identifying the cause and prescribing medications such as the local application of anesthetic, steroid ointments specifically for refractory cases, and multivitamin tablets to relieve the symptoms. Ayurvedic preparations such as Triphala oral rinse can prove to be really effective in relieving pain and burning sensation and also cause the lesions to subside, although, like other allopathy medications, it is not known to reduce the frequency of episodes. In this article, we present a case of a male patient aged 21 years who came with a complaint of ulcers on the right lateral border of the tongue. He was prescribed Triphala oral rinse and Triphala ingestion for 15 days, and on the follow-up visit, the patient reported the complete resolution of ulcers.

## Introduction

The Greek word "aphtha," which means ulcer, is where the word "aphthous" originates. Recurrent aphthous stomatitis (RAS), also known as aphthous ulcers, is a benign ulcerated lesion that is frequently found in the oral cavity. Their exact cause is unknown; however, the etiology is thought to be multifactorial. Their management is still up for debate, and their differential diagnosis calls for careful consideration and clinical expertise [1]. Three kinds of RAS are distinguished clinically: minor, major, and herpetiform. The minor form of RAS constitutes to about 70%-85%, which is the most frequent clinical presentation. The major form accounts for 7%-20% of instances and is less common.

In 5%-10% of RAS cases, the uncommon herpetiform type manifests as clusters of pinpoint ulcers that range in size from 0.1 to 0.2 cm and occur in huge numbers (5-100 ulcers simultaneously) [2]. The diagnosis is challenging because of its similarities to other mucocutaneous oral ulceration conditions. As a result, the clinical features, location, and course of the ulcerations should be taken into consideration to differentiate from other entities causing ulcers or vesiculobullous lesions [3]. RAS are difficult to manage and treat in some situations, due to their unclear etiology. Hereditary, psychological, viral, and hormonal (periods, pregnancy, or postmenopause) variables; trauma; stress; food allergies; nutritional deficiencies (iron, vitamin B12, and folic acid); and hematological abnormalities can all contribute to the development of recurrence [4]. Many efforts are made to determine the best course of treatment for this illness. Because topical medications such as anesthetic gels and steroid ointments are less likely to induce drug interactions and are both safe and effective with little side effects, they are the initial treatment of choice [5]. However, the side effects of steroids in any form cannot be ignored. There has always been an effort to prescribe such a medication, which has high effectivity with lesser side effects to improve the overall well-being of the patient. Triphala, an Ayurvedic preparation, is one such boon to treat the cases of aphthous ulcers as it possesses a wide spectrum of activity in such lesions with almost no side effects [6]. This case report discusses the efficacy of Triphala oral rinse on minor aphthous ulcers in a male patient aged 21 years.

## Case presentation

A male patient aged 21 years came to the oral medicine and radiology department with a complaint of multiple ulcers on the right lateral border of the tongue and a burning sensation in the same region for 4-5 days. The patient revealed that he had inadequate sleep and indigestion for the past 7-8 days, and also, there was a history of stress due to examination. There was difficulty in mastication and speech. The patient did not reveal any similar past episodes, and there was no history of fever before the onset of ulceration. The region with ulcers was associated with a burning sensation. His past medical and dental history was noncontributory.

**Figure 1:**
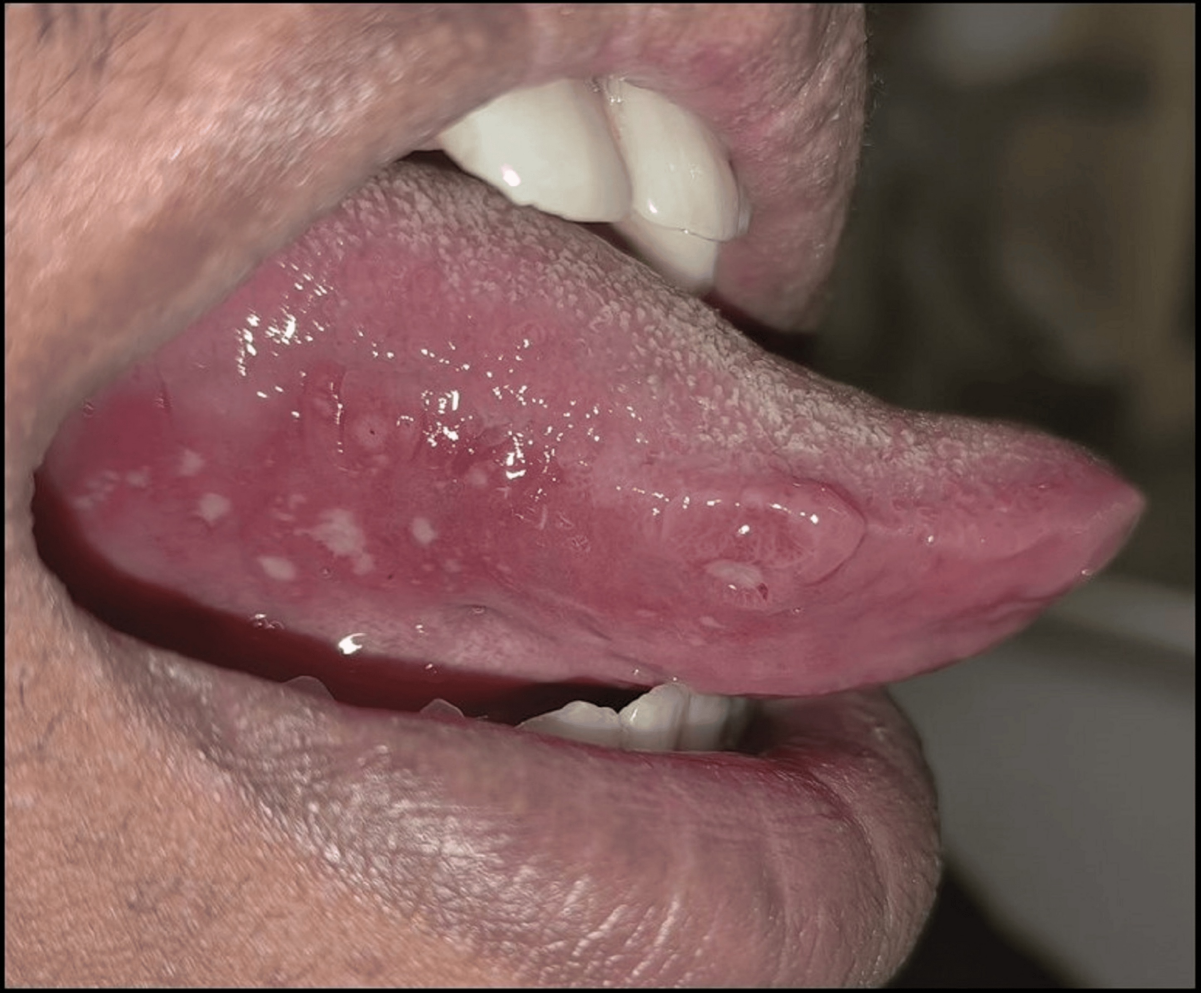
Clinical picture revealing multiple small discrete ulcers present on the right lateral border of the tongue

On careful clinical examination, multiple small ulcers were noted on the right lateral border of the tongue with sizes ranging from 0.5 × 0.5 cm to 1 × 1 cm approximately. There was a perilesional erythematous halo, and ulcers were covered with pseudomembrane (Figure 1).

There was no apparent discharge associated with ulcers, and on palpation, the ulcers were tender. Based on history and clinical features, the provisional diagnosis was made as minor aphthous ulcers.

**Figure 2:**
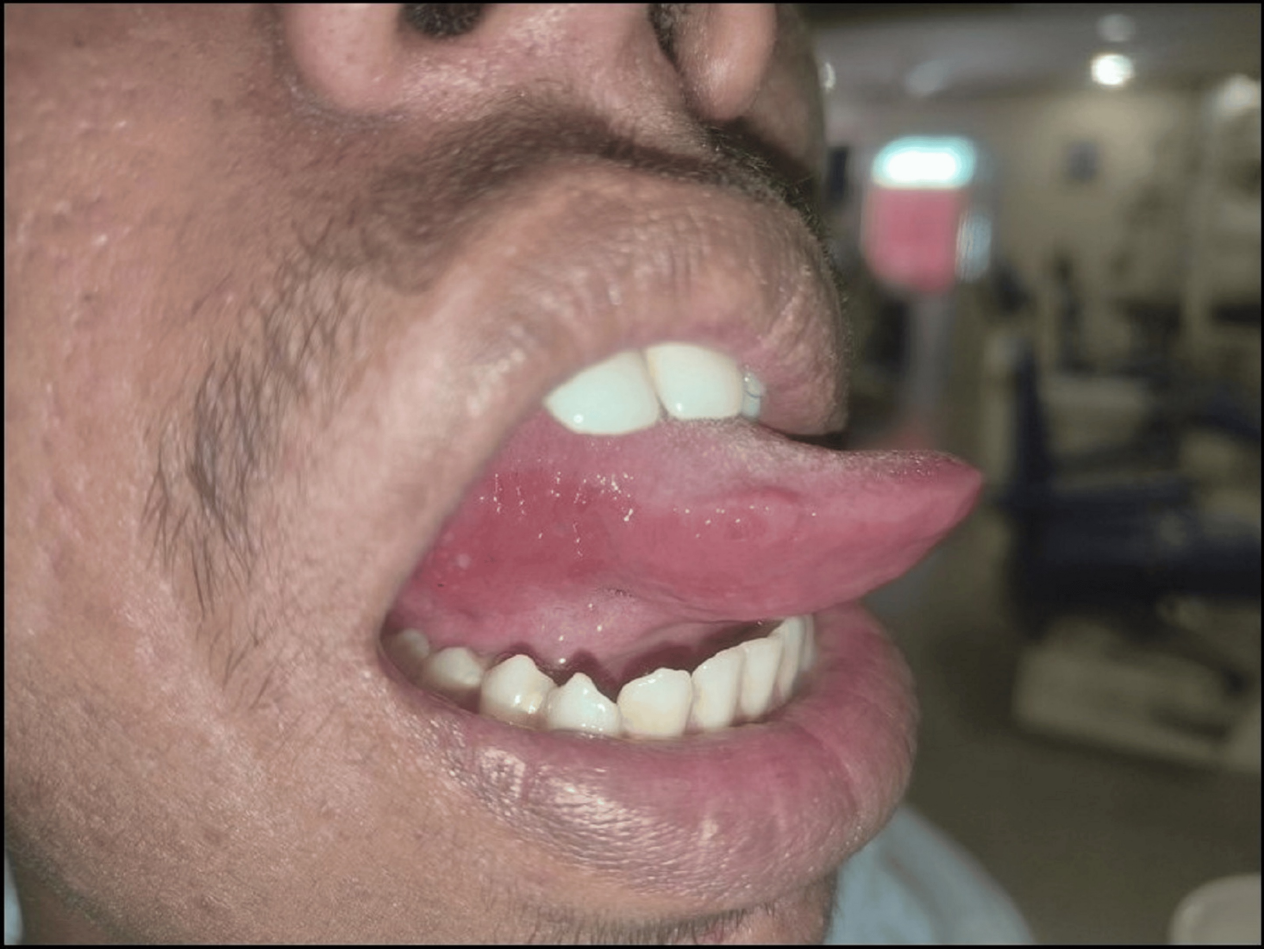
Clinical picture of the seven-day follow-up visit revealing the reduced number of ulcers and reduced erythema

As a therapeutic regimen, the patient was counseled about improving the sleep pattern. Counseling was also done for stress reduction, and the patient was advised to practice meditation, which will help to combat stress. Triphala oral rinse was advised for three times a day for seven days. The oral rinse was advised to be prepared fresh. The patient was advised to mix half a spoon of Triphala powder in half a cup of lukewarm water and stir well. Triphala ingestion was also advised once at night time after one hour of meal. On a follow-up visit after seven days, there was a reduction in the size of ulcers, and the patient revealed reduced burning sensation (Figure 2).

**Figure 3:**
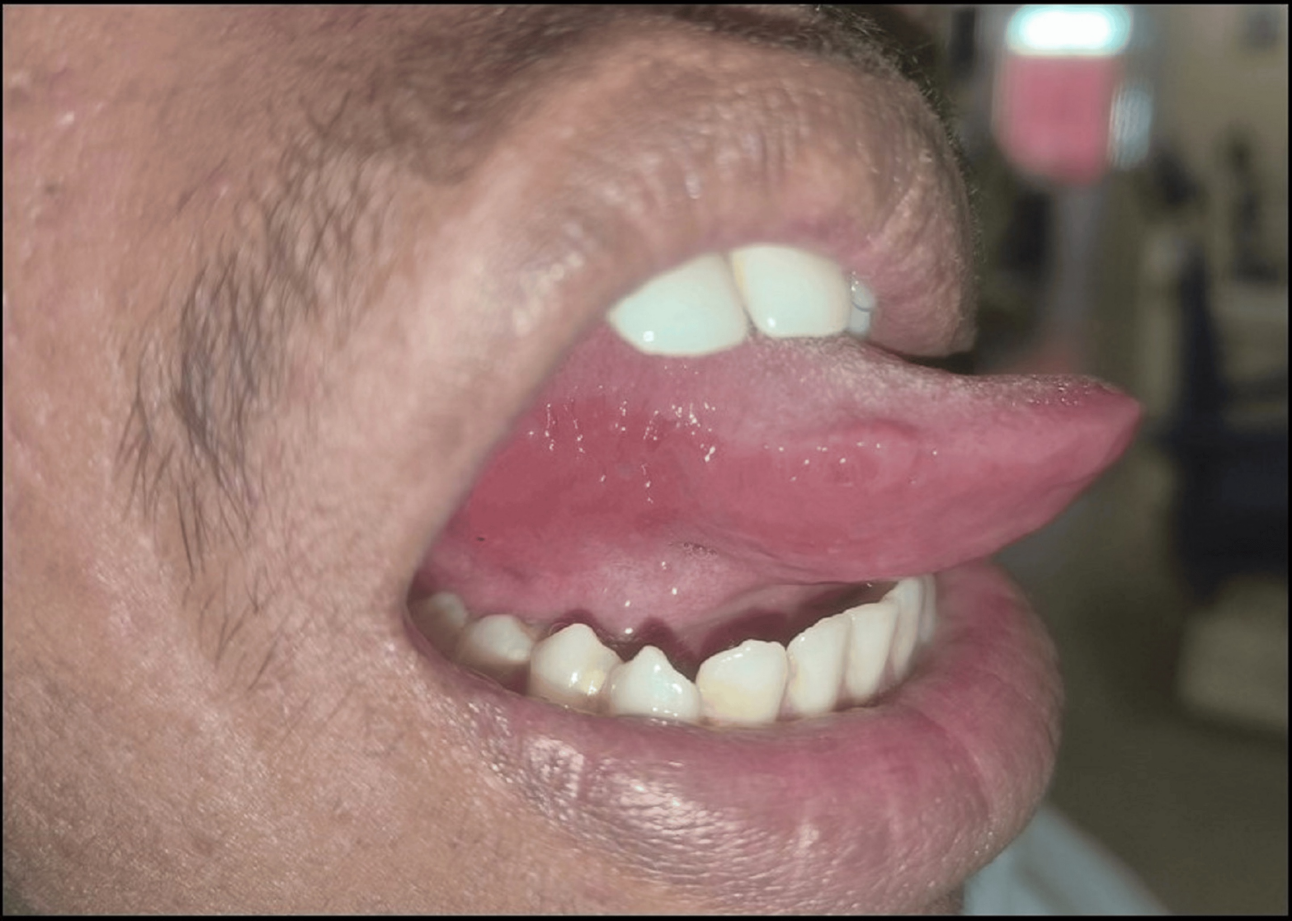
Clinical picture of the 15-day follow-up visit revealing completely resolved ulcers and erythema

For complete relief, a similar regimen was continued. On a 15-day follow-up visit, there was a complete resolution of ulcers and complete relief from the burning sensation (Figure 3).

The patient was advised regular follow-up visits in three months to keep a watch on the recurrence of the ulcers.

## Discussion

Recurrent aphthous stomatitis (RAS) is thought to be the most common mucosal lesion encountered by dentists [7]. Ulcers initially appear in childhood or adolescence and manifest as recurrent, numerous, tiny, or ovoid ulcers with yellow floors and erythematous halos surrounding them. Up to 25% of the general population suffers from aphthous ulcers, and 50% of cases reoccur within three months. Since the exact cause of aphthous ulcers is uncertain, a number of factors, including hormone fluctuations, trauma, medications, food hypersensitivity, nutritional deficiencies, stress, and tobacco use, are still thought to be associated with the disease [7]. RAS manifests clinically in three different ways: minor RAS, major RAS, and herpetiform ulceration. Minor RAS ulcers are small in size, approximately measuring less than 1 cm, and frequently occur on non-keratinized mucosa, which includes the floor of the mouth, the ventral or lateral surface of the tongue, and the labial and buccal mucosa. Major RAS ulcers are less frequent than minor ulcers and measure about 10 mm or more in diameter. These lesions are deeper and scarred, usually involving keratinized and non-keratinized mucosa, causing significant pain and dysphagia. Herpetiform ulcers constitute the least encountered entity. Herpetiform ulcers can range in size from 1 to 2 mm, and several (5-100) may appear at once. Larger confluent ulcer regions can emerge from herpetiform ulcers, frequently accompanied by noticeable redness. Lesions are extremely painful and cause severe discomfort [8].

A detailed history and examination of the ulcers are necessary for the proper diagnosis of the condition. Additionally, an external examination that includes cervical lymph node palpation is required. When evaluating a patient with oral ulceration, it is crucial to take note of their family history, frequency, duration, the number of ulcers, the site of ulcers (keratinized or non-keratinized), the size and shape of ulcers, related medical conditions, genital ulceration, skin issues, gastrointestinal disturbances, medication history, ulcer edge, ulcer base, and surrounding tissue. Other investigations may include hemoglobin and complete blood count, erythrocyte sedimentation rate/C-reactive protein, serum B12, serum/red cell folate, and antigliadin and anti-endomysial autoantibodies in individuals with chronic RAS [9].

Regarding the management of RAS, there is no consensus. Reducing symptoms, increasing disease-free intervals, and decreasing the quantity and size of ulcers are the goals of RAS treatment. The degree of disease, the past medical history of the patient, the rebout frequency, and the patient's tolerance to medications should all be considered while planning treatment for the patient [10].

Consuming enough vitamin B12 and folate might seem to be a helpful tactic in order to lessen the frequency and length of RAS episodes, according to Kozlak et al. [10]. Antimicrobial therapy along with glucocorticoids is part of the standard treatment for RAS. These drugs have been administered systemically through the oral route, as well as local application, mouthrinses, and intralesional injections. To relieve the pain, a topical anesthetic is used, such as 2% viscous lidocaine hydrochloride [11].

There are potential side effects of using steroid medication in local application, as well as systemic administration. Also, anesthetic gels can treat only locally and have no effect on the underlying cause. This can be well overcome by using Triphala, a boon of Ayurveda. "Triphala" is a well-known powdered concoction that has been utilized in Ayurveda since ancient times. Equivalent portions of *Terminalia chebula*, *Emblica officinalis*, and *Terminalia bellirica* make up Triphala. Its analgesic, antipyretic, and ulcerogenic properties were found to be similar to that of a nonsteroidal anti-inflammatory drug (NSAID), indomethacin in rat experimental models [12]. In a recent study, the efficacy of polyherbal formulation against stomach ulcers caused due to aspirin and pyloric ligation was investigated in rats [13]. Supplementing with Triphala has been demonstrated to reduce stress. A 48-day Triphala treatment can prevent cold-stress-induced behavioral and biochemical abnormalities such as an increase in immobilization, with an increase in rearing, grooming, and ambulation activity together with a notable rise in corticosterone and lipid oxidation (LPO) levels [14]. Rats protected by Triphala are less susceptible to noise-induced changes in their cell-mediated immune response and antioxidant levels. In a number of biochemical markers of inflammation, Triphala performed better than or similarly to standard pharmaceutical treatment. In rats with arthritis, Triphala also reduced inflammatory markers and cartilage and bone degradation. When it comes to reducing the symptoms of arthritis and inflammation, Triphala extract was proven to be more beneficial against those of indomethacin, an NSAID. There are no significant limitations to using Triphala for minor aphthous ulcers. However, patient compliance could be a matter of concern regarding Triphala oral rinse and ingestion due to its unpleasant taste [14].

Chour and Madni, in their clinical study in 2023, have proved that Triphala was cheap, easy to adapt, safe, and a better-choice drug in relieving all parameters of aphthous stomatitis, the results of which are comparable with the present study [15].

Nariya et al. studied the gastroprotective effect of Triphala in experimental rats with gastric ulcers and found that it shows significant protection against gastric ulcers [16].

Peterson et al., in a review article, suggested that Triphala provided protection against the negative effects of cold stress, as well as reversed the behavioral and biochemical changes brought on by stress, including elevated corticosterone and lipid peroxidation [17].

In the present study, the patient reported the complete resolution of symptoms within 15 days and also got relief from indigestion by Triphala oral rinse and ingestion.

## Conclusions

Aphthous ulcers are the most frequently encountered lesions of the oral cavity. There are multiple etiologic factors involved in the causation of aphthous ulcers such as stress, inadequate sleep, and improper digestion. Usual management strategies include the local application of anesthetic gel, steroids, and antimicrobial medications. Due to the potential side effects of these drugs, it is imperative to consider Ayurveda for the effective and holistic management of the underlying causes. Triphala, a boon to Ayurveda, has shown significant effectivity in the treatment of aphthous ulcers while also managing the possible causative factors.
